# Supplementary figures and images for: Brain stimulation competes with ongoing oscillations for control of spike timing in the primate brain
Source: PLoS Biol. 2022 May 25;20(5):e3001650. doi: 10.1371/journal.pbio.3001650 (PMC9132296; doi:10.1371/journal.pbio.3001650)

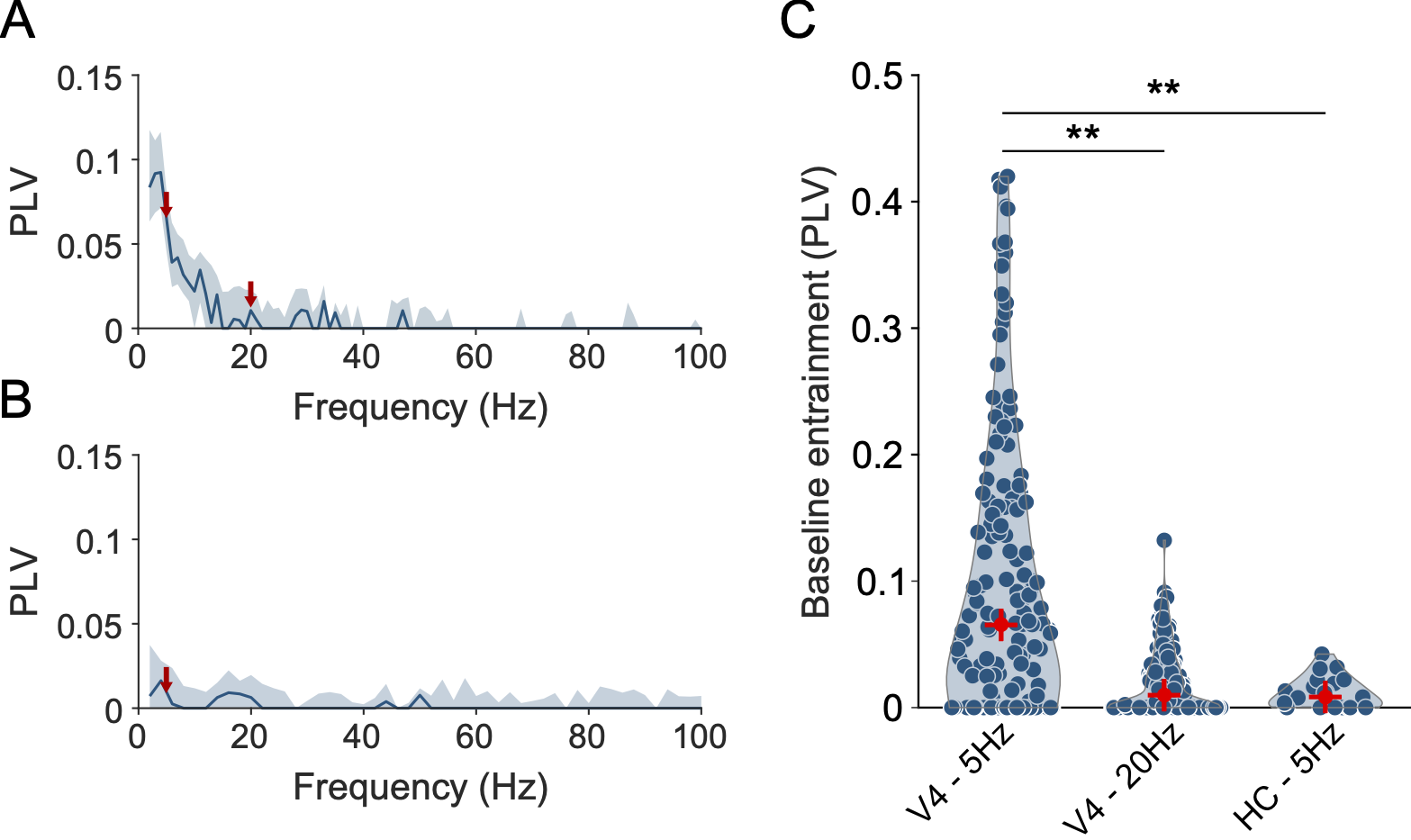

Supplement: S1 Fig — (A) Entrainment spectra for V4 neurons (N = 157), showing phase locking to the LFP in ± 1 Hz frequency bands. The median and 95% CI are shown, calculated from all V4 neurons (i.e., those used in subsequent 5 Hz and 20 Hz tACS experiments). Red arrows indicate the tACS frequencies used in those experiments. (B) Entrainment spectra for hippocampal neurons (N = 21), plotted in the same style. (C) Individual baseline values for each cell in the 3 experiment conditions. The red cross indicates the median and ** indicates significance at the p < 0.01 level. See also Fig 1; note that the V4 data are divided across panels A and B of Fig 1. Numeric values can be found in S1 Data. LFP, local field potential; tACS, transcranial alternating current stimulation. (TIFF) [file pbio.3001650.s001.tiff]

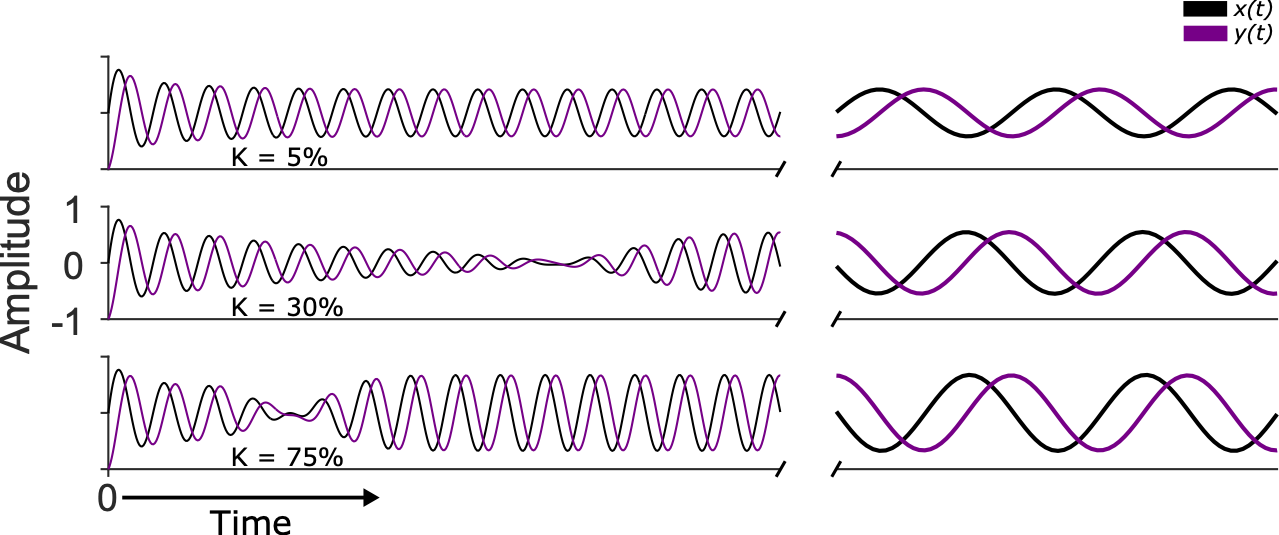

Supplement: S2 Fig — The values of x (black) and y (magenta) during 3 runs of the model with k = 5%, 30%, and 75%, demonstrating that they are phase-shifted copies of each other. Note that baseline condition is not shown here, but is shown in Fig 4. tACS, transcranial alternating current stimulation. (TIFF) [file pbio.3001650.s002.tiff]
