# Supplementary material for: Brain stimulation competes with ongoing oscillations for control of spike timing in the primate brain
Source: PLoS Biol. 2022 May 25;20(5):e3001650. doi: 10.1371/journal.pbio.3001650 (PMC9132296; doi:10.1371/journal.pbio.3001650)
Supplement: S1 Appendix — (DOCX) [file pbio.3001650.s003.docx]

Supplementary Appendix for

Brain stimulation competes with ongoing oscillations for control of spike-timing in the primate brain

Matthew R. Krause ^¶^, Pedro G. Vieira ^¶^, Jean-Philippe Thivierge and Christopher C. Pack^*^

^¶^These authors contributed equally

*Corresponding author. Email: christopher.pack@mcgill.ca

Asynchronous Quenching in Simplified Stuart-Landau Oscillators

We start with a system of two coupled Stuart-Landau oscillators

$$\begin{matrix} \frac{dx}{dt}= & \lambda x-2\pi\omega y-\gamma(x^{2}+y^{2})x+s(t) \\ \frac{dy}{dt}= & \lambda y+2\pi\omega x-\gamma(x^{2}+y^{2})y \end{matrix}$$

where *s(t)* is a periodic forcing of the form $s(t)=k\cdot sin(2\pi\omega_{s}t+\phi_{s})$, where *k* is its amplitude and $\omega_{s}$ is the frequency of the stimulation. The central result of the model presented in the paper (Fig 4) is that of “asynchronous quenching”: entrainment is maximal when the frequency of the forcing $\omega_{s}$ is closest to the intrinsic frequency of the oscillator $\omega$, but can be decreased below baseline when $\omega_{s}$ is slightly detuned instead. This result cannot be explained by a simple summation of periodic oscillations corresponding to the intrinsic and driven waves, regardless of their phase offset. To gain a mechanistic understanding of this finding, it is worthwhile to consider a simplified linear system obtained by setting $\gamma=0$, which reduces the model to

$$\begin{matrix} \frac{dx}{dt}= & \lambda x-2\pi\omega y+s(t) \\ \frac{dy}{dt}= & \lambda y+2\pi\omega x \end{matrix}$$

When $k=0$, the eigenvalues of the above expressions are $\lambda\pm i\omega$, so setting $\lambda<0$ ensures that the system is globally stable. Its dynamics are therefore easy to track analytically, but the behavior of the model is simplified: there are no reductions in entrainment as in Fig 4C and 4D. We can derive the maximal amplitude of the forced oscillator in this simplified expression (1):

$$\hat{x}\approx\frac{k\sqrt{2\pi\omega}}{\sqrt{(2\pi\omega)^{2}\Delta_{\omega}+(2\pi\omega)^{2}}}$$

where $\Delta_{\omega}=(2\pi\omega-2\pi\omega_{s})^{2}$. Here, the numerator reflects the amplitude and frequency of the forced oscillation, while the denominator takes into account the difference between the intrinsic and forced oscillation’s frequencies. As an example, consider a numerical simulation where a 2Hz forced oscillation is applied that matched the intrinsic frequency (i.e., $\omega=\omega_{s}=2$ Hz and therefore $\Delta_{\omega}=0$). In this case, the response of *x(t)* is prominent (S3 Fig, top row). However, with unmatched frequencies ($\omega_{s}=1$ Hz or $\omega_{s}\mathbf{=}3$ Hz**)**, the response is attenuated. Interestingly, similar results are obtained even when the intrinsic oscillation is left to vanish before the forced oscillation is applied (S3 Fig, bottom row). With $\lambda=-1$, Fig S3A shows agreement between the numerical and analytical results. This is robust against changes in phase, which alter the shape of the response without compromising the fit obtained from $\hat{x}$, as shown in S4B Fig. Finally, a similar distribution is obtained with $\lambda=0.1$, though in this case the system’s eigenvalues are positive and therefore the amplitude of *x(t)* is markedly higher (S4C Fig) and not amenable to the approximation described above. In all cases shown in S4 Fig, the range of forced frequencies leading to a change in the mean amplitude of *x* depends on *k*, with larger values broadening this range.

Asynchronous Quenching of Irregular Signals

The Stuart-Landau model provides a very simplified model of neural oscillations. Each population oscillates as a perfect sinusoid with no delay. Here, we show that these features are not required for quenching to occur. First, we extend the simplified model to include synaptic delay *d* between the populations:

$$\begin{matrix} \frac{dx}{dt}= & (\lambda-d)x(t)-2\pi\omega y(t-d)+s(t) \\ \frac{dy}{dt}= & (\lambda-d)y(t)+2\pi\omega x(t-d) \end{matrix}$$

As S5 Fig demonstrates, quenching occurs across a wide range of delays from 0-10 time units.

Cortical activity *in vivo* is often characterized by an irregular regime where neither the phase nor the amplitude is constant over time. Irregular activity can arise from many sources, including recurrent connectivity within local circuits as well as a common noise signal driving a neural population (2). Here, we model this activity by common additive noise:

$$\begin{matrix} \frac{dx}{dt}= & \lambda x-2\pi\omega y+s\left( t \right)+a(t) \\ \frac{dy}{dt}= & \lambda y+2\pi\omega x+a(t) \end{matrix}$$

where $a(t)$ is a band-filtered (1-5 Hz) Ornstein–Uhlenbeck process. We find a similar effect of frequency-dependent quenching, albeit noisier, as in the synchronous model. The amplitude of ongoing activity was highest when the frequency of the forced and intrinsic oscillations were matched. This result was found despite the irregular phase and amplitude of activity both prior and during the forced oscillation (S6 Fig).

Choosing parameters for quenching

The simulations in the main text (Fig 4C) show a pronounced asymmetry: stimulation at frequencies slightly below the oscillator model’s native frequency $\omega$ reduced the oscillation’s amplitude more effectively than stimulation detuned the same amount in the opposite direction (i.e., towards higher frequencies). To test whether this phenomenon occurs because of our specific choice of parameters, we fit a total of 125,000 models. These simulations included all combinations of model parameters between 0.1 – 5.0, in steps of 0.1 units. Across these simulations, the smallest value was found on the left-hand side of the peak in 93.3% to 99.7% of simulations, depending on the stimulation strength: left-hand side minima were more common and moved outwards from the center with increasing stimulation strength. Manual inspection of the remaining simulations could not rule out minima on the left-hand side, but instead suggested that the range of parameters used in the sweep did not completely capture the full shape of the frequency-amplitude curve. We also defined an asymmetry index as:

$$AI = \frac{L - R}{(L+R)/2}$$

where *L* and *R* are the smallest values to left and right of the frequency-entrainment curve’s peak. Negative AI values therefore indicate the left flank is deeper, while values near zero suggest that both flanks have similar shapes. These values were consistently negative at all stimulation intensities, as indicated by significant one-tailed Z-tests (all *p* < 0.0001).

This asymmetry also occurs in the irregular model that includes an Ornstein–Uhlenbeck process, as shown in S7 Fig. Thus, the asymmetry observed here is not due to a specific choice of the parameters. In fact, it appears to be a universal phenomenon produced by non-linear interactions between oscillators. Mondal et al. (3) report the same asymmetry in data collected from Rijke tube experiments, a model system for studying thermo-acoustic oscillations. Similar asymmetries also occur when oscillations interact in low-density gas (4) and hydrodynamic jets (5).

Asynchronous Quenching at Higher Frequencies

Frequency-dependent quenching is also observed in simulations with a higher intrinsic frequency although in this case the function relating $\Delta_{\omega}$ to the maximum amplitude of *x* is markedly broader. This occurs even when the stimulation is irregular, as shown in S8 Fig. This is an interesting prediction of the model, but additional experiments are needed to verify these predictions. This verification is critical because biophysical considerations may cause higher-frequency tACS to act slightly differently. Membrane time constants, which are not included in the Stuart-Landau model, are approximately 20 ms, approaching the period of gamma-band tACS. In our prior work, we found that 40 Hz tACS was sometimes capable of entraining irregularly-firing neurons, though the effect was slightly weaker than 5, 10, or 20 Hz stimulation (6). It is unclear what much faster frequencies might do: it has been proposed that extremely fast stimulation may be ignored by the neurons, act as DC stimulation due to some hysteresis, or impose a conduction block. Moreover, gamma oscillations are thought to be more local in origin and so both populations may receive some stimulation at the same time.


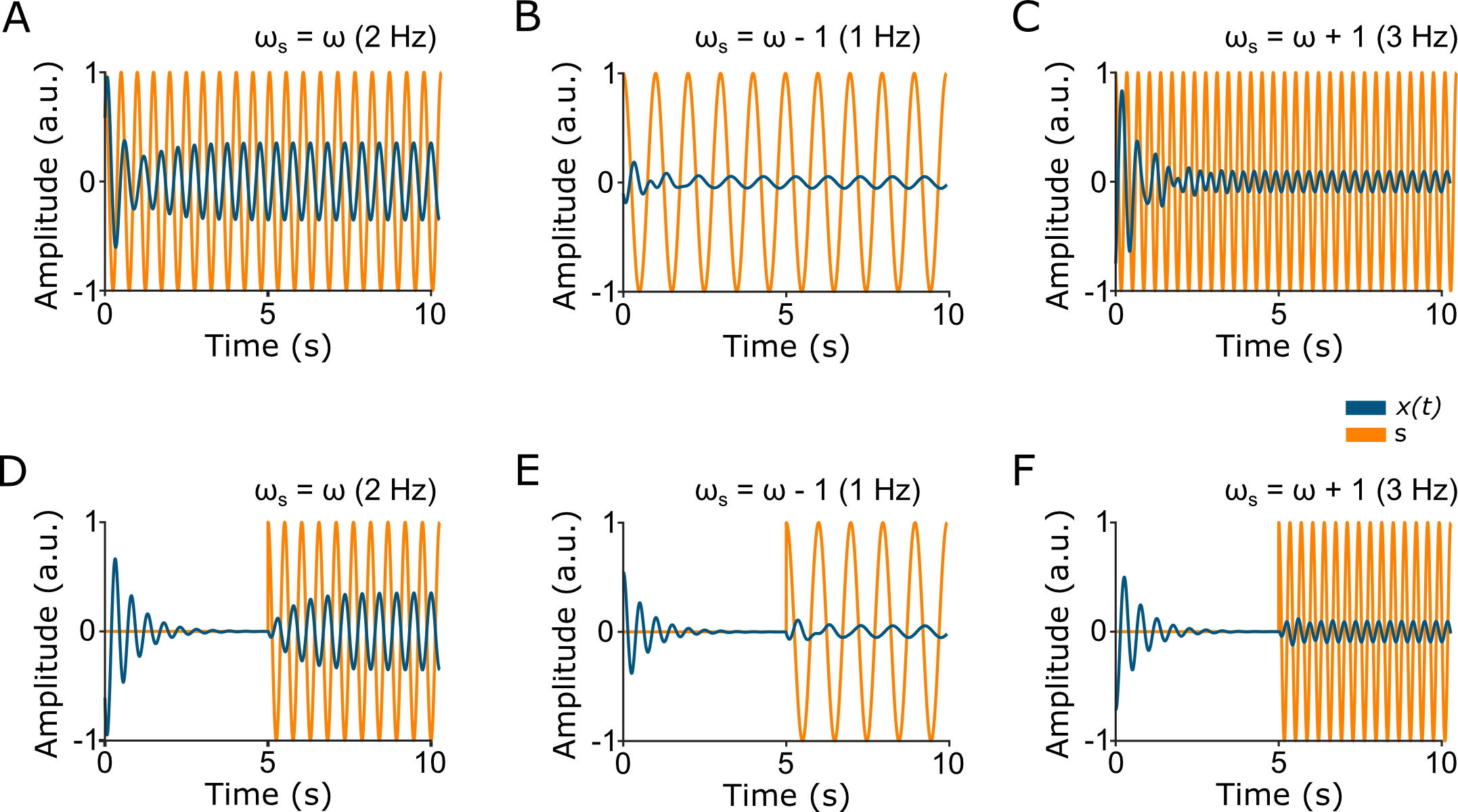


**S3 Fig. Driving a Stuart-Landau system with a forced oscillation.** In all panels, $\omega$ was set to 2 Hz while the frequency of stimulation $\omega_{s}$ was varied. **(A-C)** Stimulation (orange line) was applied to an ongoing oscillation (blue) at the same frequency (A), at a slightly lower frequency (B), and at a slightly higher frequency (C). **(D-F)** As above, except that the ongoing oscillation was allowed to decay before stimulation was applied.

**S4 Fig.** **Comparison of numerical and analytical results**. **(A-B)** Filled circles indicate the analytical solution $\hat{x}$, while the lines are derived from the numerical simulation. In all panels the intrinsic frequency was set to 5 Hz with a phase shift of the forced oscillation set to zero (A) or 1.5 radians (B). **(C)** Similar results were obtained from an unstable model where $\lambda=0.1$.

**S5 Fig. Asynchronous quenching is robust to delays between the two oscillators.** Results of simulations where the delays ranged from 1 (black) to 10 (yellow).


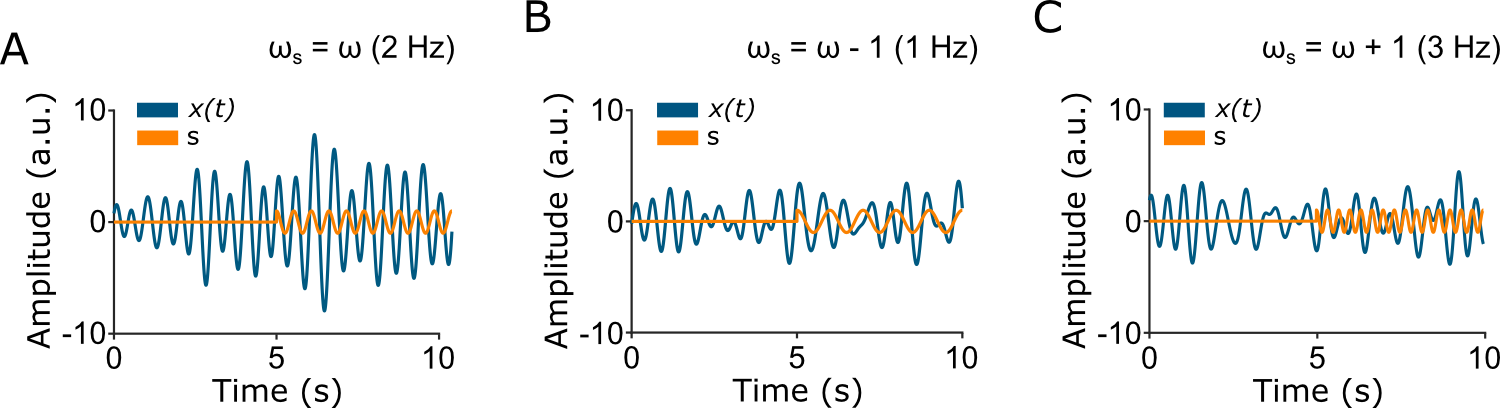


**S6 Fig. Stuart-Landau systems with irregular oscillations.** **(A-C)** Stimulation (orange line) was applied to an ongoing irregular oscillation (blue) at the same frequency (A), at a slightly lower frequency (B), and at a slightly higher frequency (C).


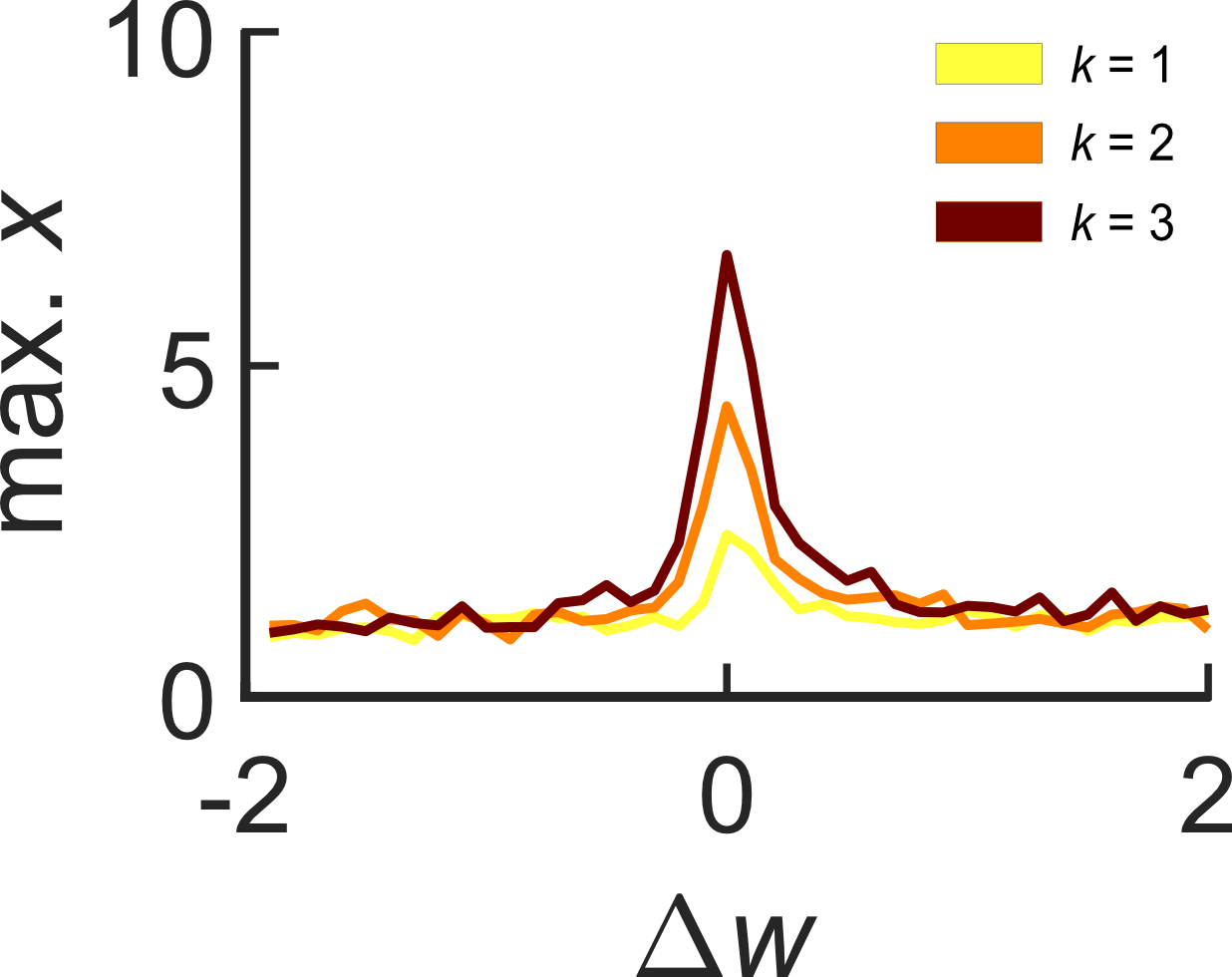


**S7 Fig. Quenching in the presence of irregular oscillations.** Results of simulations using irregular oscillations, plotted in the same style as S4 Fig and S5 Fig.

*
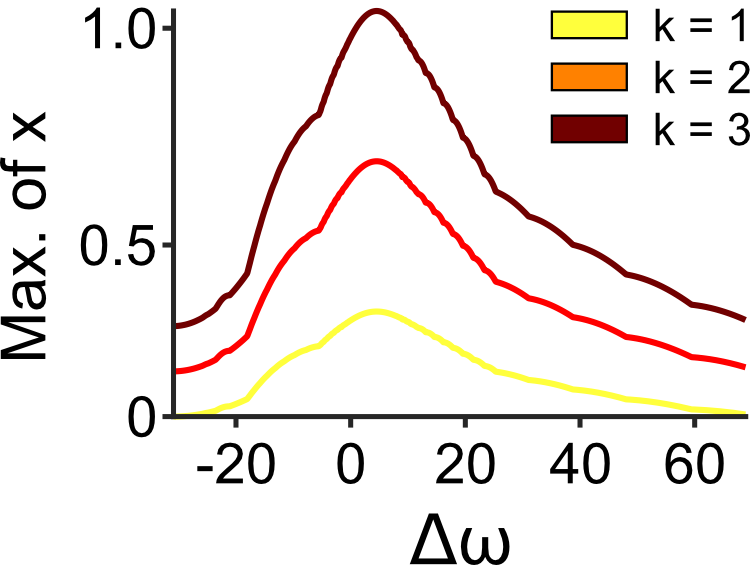
*

**S8 Fig. Quenching in the presence of irregular high-frequency oscillations.** Results of simulations using faster irregular oscillations, plotted in the same style as S4 Fig, S5 Fig, and S7 Fig.

References

1. Mori H, Kuramoto Y. Dissipative Structures and Chaos. Tokyo: Iwanami Shoten, Publishers; 1998.

2. van Vreeswijk C, Sompolinsky H. Chaos in neuronal networks with balanced excitatory and inhibitory activity. Science. 1996;274(5293):1724-6.

3. Mondal S, Pawar SA, Sujith RI. Forced synchronization and asynchronous quenching of periodic oscillations in a thermoacoustic system. Journal of Fluid Mechanics. 2019;864:73-96.

4. Hallberg MP, Strykowski PJ. Open-loop control of fully nonlinear self-excited oscillations. Physics of Fluids. 2008;20(4):041703.

5. Li LKB, Juniper MP. Lock-in and quasiperiodicity in a forced hydrodynamically self-excited jet. Journal of Fluid Mechanics. 2013;726:624-55.

6. Krause MR, Vieira PG, Csorba BA, Pilly PK, Pack CC. Transcranial alternating current stimulation entrains single-neuron activity in the primate brain. Proc Natl Acad Sci U S A. 2019;116(12):5747-55.
