# Supplementary material for: Brain stimulation competes with ongoing oscillations for control of spike timing in the primate brain
Source: PLoS Biol. 2022 May 25;20(5):e3001650. doi: 10.1371/journal.pbio.3001650 (PMC9132296; doi:10.1371/journal.pbio.3001650)
Supplement: Attachment — Submitted filename: v26 Reply to Reviewers.docx [file pbio.3001650.s006.docx]

**REVIEWS:

Reviewer #1, Robert M. G. Reinhart:**

**Summary
Transcranial alternating current stimulation (tACS) has been widely used to noninvasively modulate brain function via neural entrainment of targeted regions at a specific frequency. However, like other noninvasive brain stimulation methods, tACS receives criticisms because of its variable effects. In this article, the authors provided a promising explanation for the inconsistent effects, that is, tACS competes with the brain's ongoing oscillations. They computed phase-locking values (PLVs) that describe the consistency of spike timing and compared PLV during stimulation to baseline. The results showed that tACS modulation interacts with current brain states and successful entrainment happens when the intrinsic rhythm is weak. When neurons are strongly locked to ongoing activity, tACS works via desynchronization and re-entrainment. Model simulations demonstrated that frequency mismatch between the intrinsic oscillation and tACS stimulation is the primary cause of entrainment decrease.

I think these are novel and fascinating findings. However, some clarifications and discussion are needed to strengthen the interpretation as well as to elucidate the mechanism. Below are the concerns that should be addressed.**

Thank you for your enthusiasm! We hope that the revisions answer any lingering questions.

**Major**

**Results section, tACS alters the strength and phase of entrainment.
1. Overall, tACS altered neural activity in two dimensions: entrainment as measured by bidirectional PLV changes, and shifts of the preferred phase. Could the author discuss what's the functional relevance and difference of the two changes in terms of tACS effects on behavior?**

Both Reviewer #1 and #3 asked about the translational potential of these findings. In reviewing the literature, we were pleased to discover that similar-sized changes in both entrainment and spike phase have been associated with potentially useful outcomes. We have expanded our discussion of behavioral consequences of synchrony on p 17:

The ability to increase and decrease neuronal entrainment opens up a number of new therapeutic and experimental possibilities. Increasing the synchrony of arrhythmically-firing neurons by ~0.1 PLV may produce meaningful improvements in memory and other cognitive processes (reviewed in Krause et al., 2019). Conversely, excess synchrony has been implicated in a wide range of conditions, including movement disorders, epilepsy, and schizophrenia (Venables et al., 2009). Parkinson’s Disease, for example, creates pathological oscillations that entrain neurons with a PLV of up to 0.3 (Cagnan et al., 2019; Meidahl et al., 2019). Deep Brain Stimulation (DBS) has become a standard, albeit invasive and expensive, method for treating severe cases of some of these diseases. While the mechanisms of action are not completely understood, it seems increasingly likely that DBS acts by regulating spike timing in ways that reinstate normal patterns of activity, rather than merely suppressing pathologically strong levels of activity (Santaniello et al., 2015). Our data suggests that tACS may be capable of producing similar effects non-invasively.

The timing of spikes within an oscillatory cycle may also convey information that shapes behaviour. In rodents, spike phase relative to ongoing theta oscillations provides detailed information about the position of the animal in space. The existence and role of theta precession is still debated in primates (Courellis et al., 2019; Qasim et al., 2021); stimulation that shifts spike phase may thus provide a useful tool for investigating its role in human navigation. The phase of spiking also reflects attentional selection, both within (Masse & Cook, 2008) and across modalities (Zuo et al., 2020). These sorts of effects have been studied less frequently in humans, but our data suggests tACS may also provide a means for doing so.

In a similar vein, the Reviewer’s interest in translating results also prompted us include to an explicit suggestion for future human experiments on p. 20:

Phenomena like these may account for some of the extensive intra- and inter-subject variability observed in many tACS experiments. Since individual differences in cranial shape alter the electric fields produced by tACS, anatomical covariates have been suggested as a way to reduce this variability (Kasten et al., 2019). Our data suggests that functional covariates, such as the amplitude and peak frequency of the targeted oscillation, perhaps as measured via EEG, may be useful as well.

**2. Phase shift analysis was performed on 1) neurons without PLV changes but showed phase preference and 2) all neurons including those with no significant PLV changes. Both results suggested the occurrence of phase change irrespective of tACS entrainment. Could the authors explain the specific motivation to perform the same analysis on the sub-portion neurons without significant PLV changes? If these analyses speak to the same conclusion, why not report one of the two results and put the other one in the supplementary?**

We agree that the two analyses convey similar information, but we feel that each one by itself is vulnerable to criticism on statistical grounds. In particular, the first analysis restricts the sample to those neurons that pass an arbitrary statistical criterion for phase preferences (p < 0.05). Conceivably, one could pick a different threshold (e.g., p < 0.01) and obtain a different result, and this would be a kind of p-hacking. At the same time, analyzing all cells, including those without obvious phase preferences at baseline, could yield noisy results, which would be unbiased but perhaps unreliable. We therefore ran both analyses and were gratified to find that they yielded essentially the same answer (phase shifts of 36^o^ and 38^o^). We include both results in the paper, but in the revision we now describe the second analysis more compactly on p. 9:

Repeating this analysis using all 93 neurons where 5Hz tACS had no effect on PLV revealed a similar result: a statistically significant phase-shift towards 38 ̊ (p < 0.01; Hodges-Ajne omnibus test). The direction of these shifts is shown in the violet histogram of Fig 2A.

A vector-field plot with all 93 neurons was very difficult to read due to the overlapping lines, so we have kept the combined visualization in Figure 2.

**3. The two analyses suggest a similar phase shift toward 35.6 and 38. However, there was no explanation of why it falls in this range. Was it driven by the tACS stimulation starting phase? Second paragraph of this section (11th line) writes 'the rising phase of the tACS waveform'. Also see the last sentence in the second paragraph of Results section 'tACS first desynchronizes, then re-entrains neural activity'. Does the rising phase of tACS waveform suggest the starting phase of tACS stimulation? If yes, providing this information in Methods will solve the confusion.**

There are several different notions of time and order in our manuscript, and we apologize for the fact that they were not always clear. As we discuss below, the “first-then” section title was meant to indicate that these effects were ordered in terms of stimulus intensity, rather than time, and the wording has been revised to make that clear. However, there is another notion of order within a single stimulus cycle, and that is what we were attempting to describe here. Our stimulus consists of a continuous sine wave. Each cycle begins with a stimulation intensity of 0 (at 0˚ of phase), and the intensity increases until it reaches a peak at 90˚ of phase. It then decreases, crossing zero at 180˚ of phase and reaching its nadir at 270˚, after which it rises again towards zero and the cycle repeats. By “early, rising phase”, we meant the interval between 0—90˚ of phase, which occurs early in each tACS cycle and characterized by rising current.

The fact that tACS shifts spikes towards this part of the tACS waveform is interesting, because it is predicted by the underlying biophysics. (Radman et al., 2007) hypothesize that the filtering properties of neuronal membranes should shift spiking towards the early rising phase, with the extent of the shift depending on both the properties of the electric field and the neuron itself. They provide a derivation in the Supplement of their article (“Derivation of phase distribution for AC stimulation”) and *in vitro* data from a small number of neurons confirming it in the main text. The solution of this model, extracted from their Figure S1, is shown below.

**Supplementary Figure S1. Theoretical predictions of spike timing phase response for a**

**range of field-induced polarizations over voltage ramp slopes.** Left column: Membrane voltage over two cycles with cE/VT ranging from 0 (no AC contribution) to 1000. The cell is

constrained to fire within the range highlighted in green (see main text). Middle column: Predicted distribution of spike timing phase according to Eq. 3 (main). Right column: circular plot of phase distribution with mean angle and vector strength. The cell is constrained to fire in the green phase. The arrow direction shows the mean firing phase, relative to the extracellular field while arrow length represents vector strength, r. With increasing field strength coefficient, mean phase advances from 0 to π/2 and coherence increases. (Figure and caption from Radman et al., 2007)

Here, we show that these predictions hold up in a much more realistic *in vivo* model of tACS. We now discuss this for V4 neurons on p. 9:

These phase changes cannot be attributed to differences in referencing in the baseline and tACS conditions or to spike waveform distortions caused by tACS (see *Materials and Methods*). In fact, shifts towards this early rising phase of the tACS cycle have been predicted from biophysical properties of neuronal membranes (Radman et al., 2007).

The same physical principles should hold for the hippocampus and indeed, the data we show in Figure 3D is consistent with these predictions as well. We now mention this on p. 11 of the *Results*:

Interestingly, the preferred phase for these neurons was similar to that observed in V4 (Fig 3C). This similarity has been suggested to indicate that both populations were directly affected by tACS via the same biophysical mechanism and that the deep structure was not merely entrained by modifying its more superficial inputs (Ozen et al., 2010).

**4. S2 demonstrates the phase shift of individual neurons who had PLV increase or decrease during tACS stimulation. However, there was no information on the average phase change. Is it possible to draw a red arrow like the one in Figure 2. This will provide the opportunity to compare whether the phase shift of neurons with PLV change differs from the phase shift of neurons without PLV changes.**

We did calculate the mean phase shift for both subgroups:

- Increased PLV neurons: 206˚
- Decreased PLV neurons: 338˚

These estimates are unreliable because the populations had no overall phase preferences (p > 0.05; Hodges-Ajne omnibus test). The underlying issue is that detecting phase shifts requires conditions under which both spike phases (i.e., baseline and tACS) are well-defined. In our data, 88% of neurons (15/17) in the increased PLV group had no significant phase preference during the baseline condition, making the subtraction meaningless.

We have therefore not included these values in the revised manuscript. We have updated the caption of Figure 2 to explain why:

“No red arrows are included in these panels [Panels B and C] because the population has no net phase preference (p > 0.05; Hodges-Ajne omnibus test), in part because neurons had weak phase preferences in either the baseline condition (B) or tACS condition (C), as indicated by the clustering near the origin.”

**5. The authors introduced one novel finding of the present study in the final paragraph of the section based on S2 (tACS first decouples neural activity and then re-entrains them). I am not sure why the authors decided to put these plots in supplementary, rather than the main text. I think it might be more important than the example abcd neurons in Figure 2.**

Thank you for the suggestion. We have revised Fig 2 accordingly.

**Results section 'tACS first desynchronizes, then re-entrains neural activity'
1. The authors' interpretation of Figure 3C is that tACS competes with ongoing oscillations and exerts influence via desynchronization and re-entrain. This account seems to view the desynchronization and re-entrain process as two temporally ordered steps. I would reconsider using 'first-then' because this temporal relationship is based on independent conditions (baseline, 1mA tACS, and 2mA tACS). I am not sure why the 1mA condition (entrainment elimination) has to be the intermediate state of baseline and 2mA condition. In other words, whether desynchronization is a prerequisite for neurons with strong intrinsic oscillation when receiving intensive tACS stimulation (e.g., 2mA)?**

We agree and regret the confusion. Although our simulations do show a temporal progression (Figure 4A, B), we meant that these effects are ordered in terms of stimulation intensity: weak, frequency-mismatched stimulation leads to a net decrease in entrainment, while stronger stimulation imposes a new rhythm on the brain. The temporal order is an interesting hypothesis but difficult to address with our data. Our experiments consisted of long blocks specifically to minimize transient effects produced by the onset of stimulation (e.g., changes in arousal from the sensory percept or movement leading to cell loss). This will be an interesting avenue for future work, but for now we have changed the text to clarify this point. The final sentence of “tACS alters the strength and phase of entrainment” (*Results*, p. 9) now reads:

We therefore suggest that the phase shifts observed in the cells where PLV values were not significantly changed reflect a balanced combination of these effects: tACS has decoupled neurons from their entrainment to the ongoing oscillation and imposed a similar amount of synchronization to the stimulus waveform at a new phase.

We have changed the title of the subsequent section in the *Results* (p. 10) to:

Increasing current intensity shifts the dominant effect of tACS.

As to whether desynchronization is a pre-requisite for increased entrainment, we believe it is not. In theory, weak stimulation that was well-matched to the phase and frequency of the ongoing oscillation could immediately increase entrainment. Indeed, such effects are readily produced by our model. In the simulation below, matching the relative frequency and phase yields an immediate increase in the oscillation’s amplitude, without even a transient dip.

However, we expect that it will be extremely challenging to achieve effects like these in practice. Neural oscillations often drift in frequency and phase, even on sub-second timescales, making mismatches nearly inevitable. Stimulation will therefore generally need to overcome any pre-existing oscillation to impose its own rhythm on the neurons.

We also do not necessarily think 1 mA is always an intermediate state or transition point between desynchronization and entrainment effects. As we show in Fig 3B, some neurons experienced a partial loss of entrainment at 1 mA, followed by a more complete loss at 2 mA. We expect that these neurons would eventually become synchronized to the tACS waveform at higher currents; though we did not test this experimentally, it seems inevitable based on the underlying biophysics. As we now discuss on p. 11:

One possible interpretation of these data is that neurons vary in their susceptibility to the electric field (e.g., due to their morphology or orientation relative to the field), and that stimulation above ±2 mA would be necessary to completely desynchronize and re-entrain the cells in the latter group.

**Considering the result of model simulation in Figure 4C and how baseline frequency affects tACS effects in Figure 5, I wonder if it's possible that the entrainment decrease in 1mA tACS condition was mainly driven by frequency mismatch. The frequency mismatch issue also exists in the 2mA tACS condition, but high intensity stimulation has a larger tolerance window of frequency mismatch as shown in Figure 4C. This would raise the possibility to observe successful entrainment in the 2mA condition reflected as PLV increase in Figure 3C.**

Agreed! This is essentially the model we are proposing: stimulation competes with the ongoing oscillation. If not perfectly aligned in frequency and phase, the weaker stimulation cannot always completely overcome ongoing oscillations (Fig 3) to impose its own rhythm, and the net result of “interference” between the two oscillations is a decrease in entrainment. The stronger 2 mA stimulation sometimes—but not always—can overcome those mismatches, resulting in increased entrainment. In the limit, exceedingly strong external stimulation should completely drive the neuron regardless the extent of frequency/phase mismatch (e.g., in ECT).

**Meanwhile, how can the authors defend against an alternative explanation of Figure 3C, that is, once the tACS intensity reaches the threshold to evoke entrainment, ongoing brain activity will be immediately tuned into targeted frequency.**

Many aspects of our data are parsimoniously explained by the competition model but are difficult to square with the Reviewer’s alternative hypothesis. First, we observed partial effects on individual neurons: the cell in Fig 3B was partially desynchronized by 1 mA tACS and further desynchronized by 2 mA stimulation, suggesting a gradual shift rather than an abrupt transient between two distinct regimes. Second, we often observed reduced and increased entrainment effects on neurons recorded together during the same trial. For example, the PLV of one neuron increased from 0 during baseline to 0.10 PLV during tACS. A neuron recorded on an adjacent channel 150 µm away exhibited the opposite effect: a reduction from baseline PLV of 0.17 to ~0 during tACS. Since these effects occur simultaneously and in the presence of similar LFP signals (correlation = 0.8), they cannot be explained by brain activity locking into a common mode. Third, phase shifts are straightforward mathematical consequence of interacting sinusoids, but it is difficult to see how they might arise under the “tune-in” model. Finally, there is little evidence for a single “threshold” at which tACS becomes effective. Instead, electric fields have a continuous and graded effect on membrane potentials, as demonstrated in a number of *in vitro* experiments where these subthreshold effects can be measured directly. We believe that the apparent evidence for minimum “thresholds” reflects the statistical power of individual experimenters’ approaches rather than an underlying biological mechanism. This point is discussed in Radman et al. (2007; p. 3033):

We note that the limits to resolve a field-induced change are determined by the noise (experimental and biological) in spike timing and subsequent effect on the repeats necessary to reach statistical significance; the latter is limited by duration of neuronal impalement integrity.

We now mention the fact that cells with increased and decreased entrainment can be observed at the same time in the *Results* (p. 6-7):

This suggests that these changes in entrainment were not due to signal loss or other artifacts (Krause et al., 2019). In fact, when multiple neurons were recorded simultaneously, we often observed that tACS increased the PLV of one neuron, while decreasing the PLV of neurons recorded on an adjacent channel (150 µm apart).

Potential implications of this are discussed in the *Results* (“Implications for Biophysics”, p. 19):

As a result, the effects of fixed-frequency tACS at 10 Hz may vary both across subjects and even within a single subject. In fact, because the tACS electric field decays gradually, it may have opposing effects on different parts of the oscillatory network at the same time. We even find opposing effects on neurons recorded 150 µm apart, suggesting that the net effects of tACS may be extremely complicated and determined by both the neurons’ physical positions and their functional roles.

**2. In the last paragraph of this section, the authors mentioned 'the preferred phase for these neurons was similar to that observed in V4'. Could the author extend the discussion on why both populations showed converged preferred phase around 0 as shown in Figure 3C and 3D?**

As we described above, single-neuron biophysics predicts that neurons should shift their entrainment towards the early, rising phase of the tACS. This is particularly important because observations of similar shifts have been argued to reflect direct effects on the neurons in each area, rather than a polysynaptic effect where entrainment of superficial areas is transmitted to deeper structures (Ozen et al., 2010). We now discuss this on p. 11:

Interestingly, the preferred phase for these neurons was similar to that observed in V4 (Fig 3C). This similarity has been suggested to indicate that both populations were directly affected by tACS via the same biophysical mechanism and that the deep structure was not merely entrained by modifying its more superficial inputs (Ozen et al., 2010).

**Results section 'Similar patterns of effects emerge from a simple oscillator model'
1. In this section, the author examined the mechanism that underlies entrainment decrease and found that frequency mismatch between tACS and ongoing oscillation accounts for the findings. Figure 4C and 4D are informative demonstrations of model simulation results. I consider the relative entrainment change on the y-axis is obtained by filtering LFP at the stimulated frequency (e.g., 5Hz) and then comparing PLV between baseline and tACS condition. If this is true, I am confused why tACS would compete with ongoing oscillation at the same frequency and desynchronize it.**

Our simulations demonstrate that both tACS phase and tACS frequency differences can alter the amplitude of the ongoing oscillation. However, there is a critical difference between the phase-mismatch and frequency-mismatch effects. Phase mismatches generally just shift the phase of the oscillation. A narrow range of phase-mismatches at the same frequency do reduce the oscillation’s amplitude, but these effects are transient: the examples shown in Fig 4A are the strongest examples of phase-mismatch effects.

The plot below summarizes the behavior over time of a Stuart-Landau oscillator receiving moderate (*k=*50%) stimulation across a wide range of phase mismatches. These are normalized so that the amplitude of the unstimulated oscillator equals 1.0, with blue values indicated supressed oscillations and red denoting amplitude increases vs. baseline.

Since the phase-related desynchronization occurs briefly and only when the stimulation is almost completely out of phase, we believe it is unlikely to account for our results. In contrast, the frequency-mismatch effects reported in Fig 4C persist indefinitely, even when analyzing only data from the latter half of each run (i.e., 100-200 s).

We now discuss this on p. 12-13:

We first asked whether phase-mismatches between an ongoing oscillation and tACS at the same frequency could account for our results. They cannot. Most phase offsets do not reduce the oscillation’s amplitude, even transiently, but instead lead to a shift in phase, with the amplitude maintained or preserved. Phase offsets near 180 degrees can produce brief reductions in amplitude, but the oscillation rapidly recovers and eventually exceeds the baseline amplitude. Fig 4A shows the most extreme example of this effect. Applying completely out-of-phase stimulation (180˚ mismatch) initially depresses the oscillation’s amplitude, but these effects only last 3-6 cycles; the amplitude subsequently increases by 5%, 26%, and 49%, depending on the stimulation intensity. Since our data was collected in blocks where hundreds or thousands of tACS cycles were applied, any transient decrease in the oscillation would be overwhelmed by the subsequent long-lasting amplitude increase. Moreover, our data was collected during an open-loop stimulation paradigm, where the onset of stimulation was unrelated to the ongoing oscillation’s phase, so conditions where even these transient decreases occur were unlikely.

**2. How to interpret the asymmetry of Figure 4C that same frequency mismatch on the left side actually had a stronger effect than those on the right side?**

This interesting phenomenon appears to be a fundamental property of many systems comprised of interacting, self-excited oscillators. We conducted a large-scale simulation experiment, described below, to determine if this asymmetry depended on the specific parameters of our model. It did not. In fact, this appears to be a universal phenomenon. While some of these simpler systems permit a physical explanation of these results, we are not sure how to adapt these intuitive explanations to neural dynamics, which are less thoroughly understood.

However, one obvious implication of these results that users seeking to desynchronize activity would do better to underestimate, rather than overestimate, the target frequency, which we now mention in the *Discussion* on p. 18:

Asynchronous quenching may therefore provide a simpler and more efficient way to regulate neural activity than closed-loop systems (Mogul & van Drongelen, 2014) that are often proposed for this task, simply by applying weak tACS at a frequency slightly below the targeted oscillation (see discussion of asymmetry in the Supplemental Appendix).

As Reviewer 3 asked to minimize the amount of model-related material, we describe the parameter sweep experiment in the *Supplemental Appendix* (“Choosing Parameters for Quenching”, p. 2):

The simulations in the main text (Fig 4C) show a pronounced asymmetry: stimulation at frequencies slightly below the oscillator model’s native frequency $\omega$ reduced the oscillation’s amplitude more effectively than stimulation detuned in the opposite direction (i.e., towards higher frequencies). To test whether this phenomenon occurs because of our specific choice of parameters, we fit a total of 125,000 models. These simulations included all combinations of model parameters between 0.1 – 5.0, in steps of 0.1 units. Across these simulations, the smallest value was found on the left-hand side of the peak in 93.3 to 99.7% of simulations, depending on the stimulation strength: left-hand side minima were more common and moved outwards from the center with increasing stimulation strength. Manual inspection of the remaining simulations suggested that the range of parameters used in the sweep did not completely capture the full shape of the frequency-amplitude curve. We also defined an asymmetry index as:

$$AI = \frac{L - R}{(L+R)/2}$$

where L and R are the smallest values to left and right of the frequency-entrainment curve’s peak. Negative AI values therefore indicate the left flank is deeper, while values near zero suggest that both flanks have similar shapes. These values were consistently negative at all stimulation intensities, as indicated by significant one-tailed Z-tests (all *p* < 0.0001).

This asymmetry also occurs in the irregular model that includes an Ornstein–Uhlenbeck process, as shown in S8 Fig. Thus, the asymmetry observed here is not due to a specific choice of the parameters. In fact, it appears to be a universal phenomenon produced by non-linear interactions between oscillators. Mondal et al. (2019) report the same asymmetry in data collected from Rijke tube experiments, a model system for studying thermo-acoustic oscillations. Similar asymmetries also occur when oscillations interact in low-density gas (Hallberg & Strykowski, 2008) and hydrodynamic jets (Li & Juniper, 2013).

**3. Was entrainment change of y-axis in Figure 4C and 4D measured by amplitude (power) change or PLV change? Axis units are missing in both plots. I wonder whether y-axes in 4C and 4D used same scales?**

The outputs of the Stuart-Landau model reflect something akin to the average subthreshold activity of two coupled populations of neurons. These cannot be directly interpreted as physical units (e.g., millivolts), but demonstrate how competition can result in increased or decreased entrainment. Since the amplitude of these oscillations is in arbitrary units, we have revised Fig 4 and its caption to indicate that.

**4. Color bar in Figure 4C indicates tACS amplitude relative to baseline which is the parameter k in the first equation. This parameter determines whether one will have an entrainment increase or decrease at a given stimulation frequency (Figure 4D). Is it possible to calculate the corresponding k of 1mA and 2mA relative to baseline in the current study?**

Extracellular recordings do not permit direct measurements of the neurons’ membrane potentials, so we cannot calculate this directly from our data. A review of the literature demonstrates that membrane potentials oscillate by a few millivolts (e.g., ±2.5 mV in Bilkey and Heinemann, 1999) though larger fluctuations are observed in sleep and anesthesia. In contrast, *in vitro* experiments demonstrate that 1 V/m electric fields direct polarize neurons by approximately 1 mV. Therefore, 1 mA and 2 mA correspond *approximately* to k=40% and 80%, respectively, though the precise numbers will undoubtedly depend on the brain area, cell type, and state of the stimulated subject.

We now make this point in the *Discussion* on p. 18-19:

For example, membrane potential fluctuations of up to 2-3 mV (Bilkey & Heinemann, 1999) occur in hippocampal neurons. In contrast, the direct polarization produced by tACS generates fluctuations of < 1 mV per V/m (Radman et al., 2009; Voroslakos et al., 2018). These ratios therefore correspond to k=40% and 80%. The precise values may vary between areas and brain states—membrane potential oscillations between UP/DOWN states are much larger, for example—but many human applications seem likely to fall near the border between decreased and increased entrainment.

It is also briefly mentioned in the *Methods* (“Model Simulations”, p. 28) to explain our choice of parameters:

We tested values of *k* ranging from 0 to 100% of the baseline oscillation’s amplitude, a range which corresponds to physiologically-relevant conditions (See *Discussion**,* “Implications for human neuromodulation”).

**Minor

Figure 1A and 1B showed PLV change of example neurons. Having four examples in A but only two in B might produce the impression that tACS entrainment increment is more dominant. However, the median PLV actually decreased in tACS condition relative to baseline. If the purpose of using more examples in Figure 1A is to demonstrate the phase shift, specify it in the caption will be helpful.**

We included these to demonstrate that we could reproduce prior work showing increased entrainment during tACS—but only under specific conditions (i.e., weak ongoing oscillatory activity). As the Reviewer notes, we also wanted to demonstrate the phase shift. We have updated Fig 1’s caption to make this information more explicit:

The cells in (A) showed increased entrainment during tACS (orange) compared to baseline (blue, BL). This was sometimes (right column), but not always (left) accompanied by a shift in preferred spiking phase. However, many more neurons, like those shown in (B), had decreased entrainment during 5 Hz tACS compared to baseline.

We have also revised the *Results* section (“tACS causes bidirectional changes in spike timing”, p. 6) to make it clear that decreased entrainment was the more dominant condition:

Instead, the application of tACS led to a decrease in the phase-locking of many more V4 neurons, as shown in the two example cells of Fig 1B, whose spike times became more uniform during stimulation.

**Title Figure 1C, 1D, and 1E will make it more straightforward that they represent populations from different regions and in different frequency bands.**

Thank you for the suggestion. We have added titles to each subplot.

**Figure 1D and 1E are not referred to in the main text. I believe Results section 'Decreased entrainment is not caused by stimulation frequency or brain region' is relevant to the two plots.**

Fixed, thank you. We also refer to the (complete) Fig 1 in several places in the discussion.

**Figure 6, adding legend in 6B and 6C will make it easier to understand that purple dots represent interneurons. Again, title 6B and 6C will help to understand they demonstrate results of 5Hz and 20Hz tACS respectively. Maybe it's common in electrophysiology but the broad neuroscience audience might get confused by the subpanel in Figure 6A and 6B. Further descriptions either in the caption or in Methods are recommended.**

Done.

**Reviewer #2: Combining experimental and theoretical methods, the authors have studied the compound effect of tACS on modulating brain activity and they provided an interesting and convincing explanation for the variability of tACS effects. They found that a competition between the external tACS modulation and the internal ongoing oscillations leads to variable or even opposite effects of tACS modulation, which depends on both the strength and frequency of the tACS modulation relative to ongoing oscillations. The study is important and timely, given that tACS is a widely-used noninvasive method for modulating brain activity and behavior but its effects were not reliable or even contradictory. This study gave a clear picture for the tACS effects which were not well understood yet. The experiments, model simulation and data analysis were all carefully done and the paper was well written. There is no major concern from me on this paper, but I suggest the authors to consider the followings for further discussion to support their conclusions. I strongly support the acceptance of this paper.**

Thank you for the strong support!

**1) It is nice that the authors showed the competition between internal oscillations and external tACS regarding to their relative frequency and strength (Figure 4). It will be even better to show the final effect as a function of relative phases between the external and internal oscillations. If the internal brain oscillation is regular in frequency, then I can roughly predict the final modulatory effect depending on the relative phase; but when the brain oscillation is irregular, as several studies have shown, the phase effect might be also complicated with the consideration of oscillation frequency. The author should have some discussion about the effect from relative phases between internal and external signals. Some further simulations with the current model regarding to oscillation phases might be better (for example systematically change or randomly perturb phases of external signals).**

We agree that neural oscillations are not pure sinusoids, but instead drift in terms of phase and frequency. Consequently, we expect that mismatches are often inevitable during human tACS and tACS may therefore be expected to exert a desynchronizing effect. We now discuss this in a few places in the revised manuscript. The reviewer may also be interested in the figure on page 7 of this reply which shows the timecourse of phase-mismatch effects; frequency-mismatch effects persist indefinitely. On p. 12-13, we discuss how phase mismatches can only lead to transient suppression, while frequency mismatches cause longer-lasting effects:

We first asked whether phase-mismatches between an ongoing oscillation and tACS at the same frequency could account for our results. They cannot. Most phase offsets do not reduce the oscillation’s amplitude, even transiently, but instead lead to a shift in phase, with the amplitude maintained or preserved. Phase offsets near 180 degrees can produce brief reductions in amplitude, but the oscillation rapidly recovers and eventually exceeds the baseline amplitude. Fig 4A shows the most extreme example of this effect. Applying completely out-of-phase stimulation (180˚ mismatch) initially depresses the oscillation’s amplitude, but these effects only last 3-6 cycles; the amplitude subsequently increases by 5%, 26%, and 49%, depending on the stimulation intensity. Since our data was collected in blocks where hundreds or thousands of tACS cycles were applied, any transient decrease in the oscillation would be overwhelmed by the subsequent long-lasting amplitude increase. Moreover, our data was collected during an open-loop stimulation paradigm, where the onset of stimulation was unrelated to the ongoing oscillation’s phase, so conditions where even these transient decreases occur were unlikely.

To assess these effects under more realistic conditions, we performed additional modelling experiments using less-regular ongoing oscillations. These are described briefly in the main text on p. 13-14:

In real experiments, such a mismatch is likely unavoidable, given variability in spike timing (Softky & Koch, 1993) and peak frequencies within an oscillation (Mansouri et al., 2017), as well as the non-sinusoidal nature of neural oscillations (Burns et al., 2011; Cole & Voytek, 2017). We obtained similar quenching results, shown in the Supplemental Appendix, from simulations using Ornstein–Uhlenbeck processes, which mimics the irregular nature of real neural oscillations (van Vreeswijk & Sompolinsky, 1996).

As Reviewer 3 asked us to limit the Main Text’s modelling content, these simulations are described in more detail in the *Supplemental Appendix* (“Asynchronous Quenching of Irregular Signals, p. 2) :

Cortical activity *in vivo* is often characterized by an irregular regime where neither the phase nor the amplitude are constant over time. Irregular activity can arise from many sources, including recurrent connectivity within local circuits as well as a common noise signal driving a neural population (van Vreeswijk & Sompolinsky, 1996). Here, we model this activity by common additive noise:

$$\begin{matrix} \frac{dx}{dt}= & \lambda x-2\pi\omega y+s\left( t \right)+a(t) \\ \frac{dy}{dt}= & \lambda y+2\pi\omega x+a(t) \end{matrix}$$

where $a(t)$ is a band-filtered (1-5 Hz) Ornstein–Uhlenbeck process. We find a similar effect of frequency-dependent quenching, albeit noisier, as in the synchronous model. The amplitude of ongoing activity was highest when the frequency of the forced and intrinsic oscillations were matched. This result was found despite the irregular phase and amplitude of activity both prior and during the forced oscillation (S7 Fig).

We agree that this is an important issue and have begun examining how the phase and frequency consistency of neural signals varies across individuals, brain regions, and behavioral states. However, thoroughly characterizing these effects will need an entire paper (at least!).

**2) The competing effects were well demonstrated in this paper with a biology plausible model for brain oscillations at low frequency (such as theta, alpha or even beta), which is corresponding to their experimental data collected at 5Hz and 20Hz. I suggest to add more discussion for brain modulations at high frequency. At the conceptual level, I don't think the conclusion in this paper will be changed, but in details there might be different form of such a competition and phase-locking. When tACS goes to the modulation at high frequency (above 40Hz or in gamma band), the competing effect and its cell specificity might be different, because studies have shown that underlying mechanisms or dynamic models (for example, Jia et al. 2013 and Han et al. 2021) for brain oscillation at high frequency were different from what was used in the paper for low-frequency oscillations. I understand that the authors might not have experimental data with tACS stimulation at high frequency, but they should give some insights on what will happened when brain oscillations were generated in a different model in the discussion. This also holds for cell-specificity, because for gamma oscillations, excitatory and inhibitory neurons were coupled more tightly, but they might not be coupled (as the model in the paper showed) for brain oscillations at low frequency.**

We had focused on low-frequency oscillations, as they are a common target of human tACS studies, but it is an interesting question how they may act at higher frequencies. In our simulations, we also observed frequency-dependent quenching of gamma band activity, but the shape of the frequency-response curve was markedly broader than at low frequencies. This is a prediction of the model, but difficult to test in our current dataset because ongoing gamma oscillations were extremely weak (S1 Fig). This will be an interesting prediction to test in future experiments.

Biophysical considerations may cause higher-frequency tACS to act slightly differently. Membrane time constants are on the order of 20 ms (see a table of values extracted from the literature here: https://neuroelectro.org/ephys_prop/4/), so it is unclear what faster stimulation might do. In our prior work, we found that 40 Hz tACS was sometimes capable of entraining neurons, though the effect was slightly weaker than 5, 10, or 20 Hz stimulation. It is unclear what much faster frequencies might do: it has been proposed that extremely fast stimulation may be ignored by the neurons, act as DC stimulation due to some hysteresis, or impose a conduction block. Moreover, gamma oscillations are thought to be more local in origin and so both populations may receive some stimulation.

These differences are now mentioned in the *Discussion* (“Implications for Biophysics”, p. 20):

Our experiments focused on relatively low frequencies, which are the dominant target of tACS and persistently occur *in vivo*. However, some behavioral paradigms can elicit higher-frequency gamma oscillations, and it may be desirable to non-invasively control them with tACS. Simulations suggest that tACS may affect faster oscillations similarly, though the shape of the frequency-response curve was markedly broader (Supplemental Appendix). However, additional experiments are needed to verify these predictions because the underlying biophysics may be different: in particular, it is unclear what happens as the tACS period approaches the membrane time constant, which would be the case for gamma-band stimulation.

**Reviewer #3: This is an important study because addresses some sources of possible variability to tACS effects in humans, even touching some issues that have been not considered up to now.**

Thank you for the supportive comments.

**Results come out from two adult male rhesus monkeys: I am not an expert of monkey studies, so maybe this is a sufficient number of animals. Methodology is sound, and already published in relevant Journals.**

Most non-human primate studies use 1-2 animals. There is extensive pressure from oversight agencies (FDA and CCAC as well as local Animal Care Committees) to use the bare minimum number of monkeys needed to answer a research question. As a result of the COVID-19 pandemic, monkey prices have increased to ~$25,000 per animal in Canada and availability is extremely limited even at that price. *PLoS* *Biology* also specifically instructs authors to describe “steps taken to minimize suffering” of non-human primates. Minimizing the number of animals involved in research is one straightforward way to do so (we also provide our animals with daily enrichment, social housing, and access to a large play arena). Thus, it is not feasible for us to increase the number of animals used in this manuscript.

At the same time, we believe this sample size is sufficient for our purposes because the relevant unit of analysis in our manuscript are individual neurons, not the complete organism. Our main experiments rely on within-neuron (e.g., tACS vs. baseline) and between-neuron (e.g., pyramidal vs. interneuron) comparisons, where the sample size—in terms of spikes or cells—is sufficient to make rigorous statistical comparisons. We have tried to avoid making strong statements where the unit of analysis is the whole animal. Specifically, since electric field strength is determined by cranial anatomy, we do not claim that the electrode configurations we used will produce identical electric fields in humans—or even other monkeys. Instead, we rely on other studies which have demonstrated that the field strengths we consider here are typical, and instead focus on what those fields may do to individual neurons

This issue is discussed at length in a recent commentary by Fries and Maris (2021), who recommend publishing data even from one animal. We now mention this briefly in the *Methods* (“Quantification and Statistical Analysis”, p. 29):

Ethical and practical considerations limit the number of animals from which we can collect data. However, the critical comparisons in this paper are made within (e.g., tACS vs. baseline) and between (e.g., putative pyramidal vs. interneuron) individual neurons, making the cell rather than the animal the relevant unit of analysis (Fries & Maris, 2021).

**However, as it stands the manuscript is extremely long and, overall, a bit hard to read even for a specialist of the field. One possibility to reduce this problem is to take off all the modeling part, that has by itself the potential for a separate (theoretical) work. In this way, moreover, there is no risk to interpret modelling results (for example those on tACS effects on different cell types) as consequence of the physiological experiment.**

The main text is ~5000 words, the length of a standard research article. The *Methods* section is admittedly quite long, but we have tried to structure the *Results* so that a typical reader can understand the paper without constantly referring to it. At the same time, we also feel is important to fully describe the myriad methods needed to collect and interpret this data. We believe the modelling component provides an important framework for understanding these data, and the editor’s decision letter suggested that we retain it. To streamline the paper, we have moved some of the newly-requested analyses to the Supplemental Appendix and revised the presentation of the model.

We do want to clarify, however, that the effects on different cell types occurred *both* in the model and in our empirical data. Model output is only shown in Fig 4; all other results are derived from our neural recordings. We have made several minor edits to the text to make this clearer.

For example, on p. 14 (*Results*, “Baseline frequency preference determines the effects of tACS”):

We tested this prediction in our experimental data by identifying each V4 neuron’s baseline frequency preference.

**One issue that is not properly addressed and should be better conceptualized and discussed is the relationship between spike activity of a single neuron (i.e., the main focus of the current study), the overall firing rate (whose changes have been never observed in this study, according to Fig. 1 and text) and the complex oscillatory networks that are the target of human tACS studies. This seems a crucial point for fully appreciating the potential translational relevance of the observed effects. I presume, for example, that a change of spiking in a small subset of neurons belonging to a discrete cortical region could be entirely shadowed (either "electrically" and, of course, behaviorally) within a distributed oscillatory activity of a complex network.**

We disagree slightly with the reviewer’s premise that small changes in neural activity cannot have appreciable effects on behavior. Rats can detect stimulation of a single neuron in barrel cortex (Houweling & Brecht, 2008). Producing a single additional action potential in ~300 neurons also causes detectable effects on an animal’s behavior (Huber et al., 2008). Small, sparse changes in brain activity can therefore have profound effects on behavior, as reviewed in (Wolfe et al., 2010). Epilepsy is a particularly extreme example of this: the aberrant activity within a small seizure focus is not shadowed by other ongoing activity, but eventually propagates throughout the brain.

Although tACS provides subthreshold, rather than suprathreshold, input to neurons, it also stimulates many, many more cells: the focus of stimulation covers at least a centimeter of cortex in humans, even “high-definition” electrode montages; sponge electrodes stimulate an even larger area, albeit more diffusely. Prior work had suggested that tACS only affects a small subset of neurons within that region. One contribution of the present manuscript is to demonstrate that tACS affects many more neurons when PLV increases/decreases and phase shifts are taken into account.

We describe this in the *Discussion* (“Implication for Biophysics”, p. 21):

Previous work has also suggested that tACS affects only a small subset of neurons. For example, Johnson et al. (2020) reported that tACS alters PLVs in 9 to 27 percent of neurons, depending on the stimulation intensity. Likewise, Ozen et al. (2010) reported that less than 30 percent of neurons were entrained by fields of ~1 V/m, and our previous work also found tACS-induced PLV changes in only a third to a half of neurons (Krause et al., 2019; Vieira et al., 2020). However, these reports considered only individually significant net changes in PLV. The diverse effects demonstrated in Figure 2 suggest that tACS may affect many more neurons by increasing PLV, decreasing PLV, or shifting the phase of entrainment. Thus, experimental constraints and the choice of metrics has led the field to overestimate the sparseness of tACS’s effects.

We therefore think that it is unlikely that these effects are completely “shadowed” by ongoing neural activity. On the other hand, we completely agree with the reviewer that the interplay of ongoing brain activity and tACS will have complicated effects on behavior. In fact, because the tACS-induced electric fields gradually decay outwards from the focus of stimulation, their effects on a distributed network are likely to be extremely complex: areas receiving strong stimulation may become more entrained, while the surrounding areas receiving less become desynchronized. In some situations, this may be beneficial (e.g., it seems akin to focusing attention), but in others it may potentially blunt the effect of stimulation. We thank the reviewer for this interesting suggestion, which will motivate some of our future work.

We now discuss this possibility in the *Discussion* (p. 19) in general terms:

More generally, our results suggest that the effects of tACS likely depend on the behavioral task, insofar as many behaviors modulate the strength of ongoing neural oscillations. Modulating activity in the 8–12 Hz “alpha” frequency band is a very common use of tACS, but the dominant frequency of endogenous alpha oscillations varies across subjects due to age, sex, clinical status, and other factors (e.g.,` Chiang et al., 2011; Mathewson et al., 2012). Within a single subject, task demands, and overall alertness of the subject alter alpha oscillations’ amplitude (Jensen et al., 2002; Klimesch, 2012; Okuhata et al., 2013). As a result, the effects of fixed-frequency tACS at 10 Hz may vary both across subjects and even within a single subject. In fact, because the tACS electric field decays gradually, it may have opposing effects on different parts of the oscillatory network at the same time. We even find opposing effects on neurons recorded 150 µm apart, suggesting that the net effects of tACS may be extremely complicated and determined by both the neurons’ physical positions and their functional roles.

**In Fig. 2 I would modify the colors of the baseline and tACS dots: now they appear as blue and black, respectively, and it is not so easy to distinguish them in the figure.**

Thank you for the suggestion. We changed the dot sizes and colors to make them easier to distinguish.

# References

Bilkey, D. K., & Heinemann, U. (1999). Intrinsic theta-frequency membrane potential oscillations in layer III/V perirhinal cortex neurons of the rat. *Hippocampus, 9*(5), 510-518. doi:10.1002/(SICI)1098-1063(1999)9:5<510::AID-HIPO4>3.0.CO;2-9

Burns, S. P., Xing, D., & Shapley, R. M. (2011). Is gamma-band activity in the local field potential of V1 cortex a "clock" or filtered noise? *J Neurosci, 31*(26), 9658-9664. doi:10.1523/JNEUROSCI.0660-11.2011

Cagnan, H., Mallet, N., Moll, C. K. E., Gulberti, A., Holt, A. B., Westphal, M., . . . Sharott, A. (2019). Temporal evolution of beta bursts in the parkinsonian cortical and basal ganglia network. *Proc Natl Acad Sci U S A, 116*(32), 16095-16104. doi:10.1073/pnas.1819975116

Chiang, A. K., Rennie, C. J., Robinson, P. A., van Albada, S. J., & Kerr, C. C. (2011). Age trends and sex differences of alpha rhythms including split alpha peaks. *Clin Neurophysiol, 122*(8), 1505-1517. doi:10.1016/j.clinph.2011.01.040

Cole, S. R., & Voytek, B. (2017). Brain Oscillations and the Importance of Waveform Shape. *Trends Cogn Sci, 21*(2), 137-149. doi:10.1016/j.tics.2016.12.008

Courellis, H. S., Nummela, S. U., Metke, M., Diehl, G. W., Bussell, R., Cauwenberghs, G., & Miller, C. T. (2019). Spatial encoding in primate hippocampus during free navigation. *PLoS Biol, 17*(12), e3000546. doi:10.1371/journal.pbio.3000546

Fries, P., & Maris, E. (2021). What to do if N is two? *arXiv preprint arXiv:2106.14562*.

Hallberg, M. P., & Strykowski, P. J. (2008). Open-loop control of fully nonlinear self-excited oscillations. *Physics of Fluids, 20*(4), 041703. doi:10.1063/1.2908008

Houweling, A. R., & Brecht, M. (2008). Behavioural report of single neuron stimulation in somatosensory cortex. *Nature, 451*(7174), 65-68. doi:10.1038/nature06447

Huber, D., Petreanu, L., Ghitani, N., Ranade, S., Hromadka, T., Mainen, Z., & Svoboda, K. (2008). Sparse optical microstimulation in barrel cortex drives learned behaviour in freely moving mice. *Nature, 451*(7174), 61-64. doi:10.1038/nature06445

Jensen, O., Gelfand, J., Kounios, J., & Lisman, J. E. (2002). Oscillations in the alpha band (9-12 Hz) increase with memory load during retention in a short-term memory task. *Cereb Cortex, 12*(8), 877-882. doi:10.1093/cercor/12.8.877

Johnson, L., Alekseichuk, I., Krieg, J., Doyle, A., Yu, Y., Vitek, J., . . . Opitz, A. (2020). Dose-dependent effects of transcranial alternating current stimulation on spike timing in awake nonhuman primates. *Sci Adv, 6*(36). doi:10.1126/sciadv.aaz2747

Kasten, F. H., Duecker, K., Maack, M. C., Meiser, A., & Herrmann, C. S. (2019). Integrating electric field modeling and neuroimaging to explain inter-individual variability of tACS effects. *Nat Commun, 10*(1), 5427. doi:10.1038/s41467-019-13417-6

Klimesch, W. (2012). alpha-band oscillations, attention, and controlled access to stored information. *Trends Cogn Sci, 16*(12), 606-617. doi:10.1016/j.tics.2012.10.007

Krause, M. R., Vieira, P. G., Csorba, B. A., Pilly, P. K., & Pack, C. C. (2019). Transcranial alternating current stimulation entrains single-neuron activity in the primate brain. *Proc Natl Acad Sci U S A, 116*(12), 5747-5755. doi:10.1073/pnas.1815958116

Li, L. K. B., & Juniper, M. P. (2013). Lock-in and quasiperiodicity in a forced hydrodynamically self-excited jet. *Journal of Fluid Mechanics, 726*, 624-655. doi:10.1017/jfm.2013.223

Mansouri, F., Dunlop, K., Giacobbe, P., Downar, J., & Zariffa, J. (2017). A Fast EEG Forecasting Algorithm for Phase-Locked Transcranial Electrical Stimulation of the Human Brain. *Front Neurosci, 11*, 401. doi:10.3389/fnins.2017.00401

Masse, N. Y., & Cook, E. P. (2008). The effect of middle temporal spike phase on sensory encoding and correlates with behavior during a motion-detection task. *J Neurosci, 28*(6), 1343-1355. doi:10.1523/JNEUROSCI.2775-07.2008

Mathewson, K. J., Jetha, M. K., Drmic, I. E., Bryson, S. E., Goldberg, J. O., & Schmidt, L. A. (2012). Regional EEG alpha power, coherence, and behavioral symptomatology in autism spectrum disorder. *Clin Neurophysiol, 123*(9), 1798-1809. doi:10.1016/j.clinph.2012.02.061

Meidahl, A. C., Moll, C. K. E., van Wijk, B. C. M., Gulberti, A., Tinkhauser, G., Westphal, M., . . . Sharott, A. (2019). Synchronised spiking activity underlies phase amplitude coupling in the subthalamic nucleus of Parkinson's disease patients. *Neurobiol Dis, 127*, 101-113. doi:10.1016/j.nbd.2019.02.005

Mogul, D. J., & van Drongelen, W. (2014). Electrical control of epilepsy. *Annu Rev Biomed Eng, 16*, 483-504. doi:10.1146/annurev-bioeng-071813-104720

Mondal, S., Pawar, S. A., & Sujith, R. I. (2019). Forced synchronization and asynchronous quenching of periodic oscillations in a thermoacoustic system. *Journal of Fluid Mechanics, 864*, 73-96. doi:10.1017/jfm.2018.1011

Okuhata, S., Kusanagi, T., & Kobayashi, T. (2013). Parietal EEG alpha suppression time of memory retrieval reflects memory load while the alpha power of memory maintenance is a composite of the visual process according to simultaneous and successive Sternberg memory tasks. *Neurosci Lett, 555*, 79-84. doi:10.1016/j.neulet.2013.09.010

Ozen, S., Sirota, A., Belluscio, M. A., Anastassiou, C. A., Stark, E., Koch, C., & Buzsaki, G. (2010). Transcranial electric stimulation entrains cortical neuronal populations in rats. *J Neurosci, 30*(34), 11476-11485. doi:10.1523/JNEUROSCI.5252-09.2010

Qasim, S. E., Fried, I., & Jacobs, J. (2021). Phase precession in the human hippocampus and entorhinal cortex. *Cell, 184*(12), 3242-3255 e3210. doi:10.1016/j.cell.2021.04.017

Radman, T., Ramos, R. L., Brumberg, J. C., & Bikson, M. (2009). Role of cortical cell type and morphology in subthreshold and suprathreshold uniform electric field stimulation in vitro. *Brain Stimul, 2*(4), 215-228, 228 e211-213. doi:10.1016/j.brs.2009.03.007

Radman, T., Su, Y., An, J. H., Parra, L. C., & Bikson, M. (2007). Spike timing amplifies the effect of electric fields on neurons: implications for endogenous field effects. *J Neurosci, 27*(11), 3030-3036. doi:10.1523/JNEUROSCI.0095-07.2007

Santaniello, S., McCarthy, M. M., Montgomery, E. B., Jr., Gale, J. T., Kopell, N., & Sarma, S. V. (2015). Therapeutic mechanisms of high-frequency stimulation in Parkinson's disease and neural restoration via loop-based reinforcement. *Proc Natl Acad Sci U S A, 112*(6), E586-595. doi:10.1073/pnas.1406549111

Softky, W. R., & Koch, C. (1993). The highly irregular firing of cortical cells is inconsistent with temporal integration of random EPSPs. *J Neurosci, 13*(1), 334-350. Retrieved from https://www.ncbi.nlm.nih.gov/pubmed/8423479

van Vreeswijk, C., & Sompolinsky, H. (1996). Chaos in neuronal networks with balanced excitatory and inhibitory activity. *Science, 274*(5293), 1724-1726. doi:10.1126/science.274.5293.1724

Venables, N. C., Bernat, E. M., & Sponheim, S. R. (2009). Genetic and disorder-specific aspects of resting state EEG abnormalities in schizophrenia. *Schizophr Bull, 35*(4), 826-839. doi:10.1093/schbul/sbn021

Vieira, P. G., Krause, M. R., & Pack, C. C. (2020). tACS entrains neural activity while somatosensory input is blocked. *PLoS Biol, 18*(10), e3000834. doi:10.1371/journal.pbio.3000834

Voroslakos, M., Takeuchi, Y., Brinyiczki, K., Zombori, T., Oliva, A., Fernandez-Ruiz, A., . . . Berenyi, A. (2018). Direct effects of transcranial electric stimulation on brain circuits in rats and humans. *Nat Commun, 9*(1), 483. doi:10.1038/s41467-018-02928-3

Wolfe, J., Houweling, A. R., & Brecht, M. (2010). Sparse and powerful cortical spikes. *Curr Opin Neurobiol, 20*(3), 306-312. doi:10.1016/j.conb.2010.03.006

Zuo, Y., Huang, Y., Wu, D., Wang, Q., & Wang, Z. (2020). Spike Phase Shift Relative to Beta Oscillations Mediates Modality Selection. *Cereb Cortex, 30*(10), 5431-5448. doi:10.1093/cercor/bhaa125
